# The Dental Profession in Relation to the General Health

**Published:** 1902-06

**Authors:** Alexander Macleod

**Affiliations:** Boston, Mass.


### THE DENTAL PROFESSION IN RELATION TO THE GENERAL HEALTH.

BY ALEXANDER MACLEOD, D.D.S., BOSTON, MASS.

In the days of the ancient Greeks and Romans, when the gods of mythology received the homage of the cultured and the learned, Æsculapius, the god of medicine, was honored with profound respect. And down through the channel of the centuries the art of healing by the practical application of the knowledge of medicine has been a potent factor in the welfare of the human race. From the comparative crudeness of early times the evolution of surgery can be traced through its vicissitudes, until to-day it has become almost a distinct science, overcoming and ameliorating many of the ills to which humanity is heir. Of a comparatively recent conception is the profession of dentistry, although we do not claim that it was either unknown or unpractised by the ancients. It is a natural product of the great dual science of medicine and surgery, combining many of the elements of both, and daily becoming more recognized by the professions and the laity as an ever-broadening scientific specialty. When we consider that the history of dentistry in America, as a specialty, brings us back scarcely more than half a century, the progress that has been made is abundant evidence that the world requires the service which it gives.

In America was established the first institution devoted exclusively to instruction in dental science; and in America the profession in all its branches has made the greatest progress. The extraordinary advancement witnessed in recent years, however, is not due so much to improvement in technic as to the extending of the field of dental education to embrace not only a study of the physiological menaces to the general health originating in the oral cavity, but also in exacting from the dentist, by the higher institutions of learning, a more general knowledge of the nature of diseases outside the sphere of his profession. In this way is he not only enabled to exercise greater personal protection, but to extend the benefit of this knowledge to all with whom he comes in professional contact who may stand in need of the same.

The greater length of time generally required for his ministrations often brings the dentist in closer relations with his patients than is either the physician or the surgeon. The results of his labor oftentimes go towards restoring to normality unsightly defects of nature which interfere with the charms of natural attractiveness. The consequent intimacy of interests thereby created, —on the one hand, the satisfaction of realizing the successful application of professional skill; on the other, the grateful appreciation of beneficial results,—begetting mutual confidence, make it easier for the dentist to study idiosyncrasies, which may lead to the discovery of obscure symptoms of disease that might easily escape the notice of the family physician.

In the minds of the greater number of even the intelligent laity the physician and surgeon are looked upon as a resource to be considered contemporary with illness, while dental operations are generally performed while in comparative health; thus, invaluable advice may be offered in special cases, with less liability to embarrassment, which would give the physician earlier opportunity to combat impending physiological derangements. From this it is not to be inferred that an invasion of the territory of the medical practitioner is suggested or approved, but to demonstrate the far-reaching effect that the tendency to widen the scope of dental education may have in aiding to promote the general health.

The mouth is the great portal through which enters not only the means by which life is sustained, but also the greater number of the germs which act to assail the integrity of the constitution and the welfare of the human organisms; and on the physiological ability of the organisms to withstand the assaults of these germs depends the health of the individual.

Digestion is one of the most important functions entering into a consideration of the bodily health, for the great design of the food we take is to give nourishment to the body; on digestion, therefore, by which the food is reduced to a soluble condition, depend the absorption, circulation, and assimilation of this nourishment necessary to support the various tissues. Under normal conditions these operations are conducted silently and harmoniously, with marvellous delicacy and completeness, and without that friction and consequent loss of power which attend the working of the most perfect machinery of man's invention.

The first step in this all-important process begins in the mouth, and proper mastication is a most essential factor in the continuation of normal conditions. This preliminary function will appear the more important when we reflect that it is the only one which we can regulate by the will, as the subsequent acts of digestion are involuntary and unconsciously performed.

Nature has endowed humanity with perfect means towards the end of natural mastication, but the nature of the food materials, which the teeth are designed to work upon, render the strictest attention necessary; and the skill of the dentist is invariably required in order that their integrity may be maintained or restored. In the famous history of Don Quixote, the hero of La Mancha, it is related that at the end of one of his great battles, wherein he was, as usual, conquered, he found himself wounded in the face by a violent blow from a stone, and grieved to find that with it he had lost one of his teeth. Reflecting a while on this unhappy accident, he sagely remarked that to lose a molar was very much like losing an old friend. And it is an important matter, in view of their relation to the general health, to see to the welfare of the molars, that they may really become *old friends.*

It is manifestly impossible here to enter into even a superficial generalization of the ill-effects accruing from neglect of the teeth and general oral hygiene. Some of the most distinguished stomatologists and bacteriologists in Europe, as well as in this country, have frequently called attention to the fact that septic conditions of the oral cavity are etiologic factors in many diseases of the general system. It is not at all uncommon to be able to trace directly to the unhealthy conditions of the mouth and the teeth local diseases, as of the eyes, ears, nasal cavities, or vocal organs; while in not a few cases the mucous membranes are deranged from the lips throughout the alimentary tract. Certain constitutional diseases of the most virulent type are primarily manifested here, and precautionary knowledge is an absolute essential of the higher duties of the practitioner to his patient. "In relation to the whole group of internal conditions caused by pyogenic organisms there is a wide field of preventive medicine open by the exercise of oral antisepsis, a field that can be worked in with the most surprisingly satisfactory results, alike by the physician, the surgeon, the dentist, and the patient." The great responsibility of the dentist, in relation to the general health of the latter, becomes patent to the most casual observer; not in treatment only, but in educating the patient to a higher appreciation of the principles of modern antisepsis.

In the administration of anæsthetics, when prolonged operations are necessary, it is as incumbent upon the dentist to first ascertain the physical condition of the patient as it is upon the physician or the surgeon. Such examination may reveal lesions of heart, lung, kidney, etc., of which the patient may be entirely unconscious; and the knowledge and advice of the dentist may here suggest the "ounce of prevention," which with prompt attention may arrest in the primary stage that which would develop serious consequences. Procrastination is an inherent quality of human nature, but the intelligent mind is quickly aroused to the importance of promptly encompassing self-preservation.

"We have no scales by which we can weigh our faithfulness to duties, or determine their relative importance in God's eyes." That which sems a trifle to us may be the secret spring which shall move the issues of life and death.

The time is long past for considering dentistry from a mechanical stand-point alone. This is an age of specialties, and dentistry is a particular specialty of that study which embraces the whole human system. This is being recognized by the foremost institutions of the country; and the higher education of the dentist, by the general extension of the curriculum, is receiving the attention which its importance deserves. The goal of human achievement was never so high as to-day.

Always the future grows out of the past, but we are here to shape that future, and we must shape it intelligently, wisely, efficiently. There is no limit to the good that well-equipped professional men and women can do save the limit they themselves make by their lack of faith in God and man, God's image. In the words of Hamlet, "The readiness is all."

